# Phospholipase A2 Isoforms in Lung Immunity and Respiratory Infections: Potential Targets for Next-Generation Therapy

**DOI:** 10.3390/ijms27114740

**Published:** 2026-05-25

**Authors:** Shweta Joshi, Kelly Walter, Dante Hamiel, Divyasha Saxena, Jian Zheng

**Affiliations:** 1 Institute of Health Management Research, IIHMR University, Prabhu Dayal Marg, Jaipur 302029, India; shweta26jan83@gmail.com; 2 Department of Microbiology and Immunology, School of Medicine, University of Louisville, Louisville, KY 40202, USA; kelly.walter@louisville.edu (K.W.); dantehamiel@yahoo.com (D.H.); 3 Center for Predictive Medicine, School of Medicine, University of Louisville, Louisville, KY 40202, USA

**Keywords:** phospholipase, immunity, respiratory infection

## Abstract

Despite the critical role of lipid-mediated signaling in regulating host immunity, endorsed by growing evidence, the interaction between lipid metabolism and immune response remains largely unknown. This review aims to elucidate the immunomodulatory role of a lung-enriched lipid metabolic pathway mediated by the phospholipase A2 (PLA_2_) family, which comprises a diverse range of lipid-hydrolyzing enzymes. Based on their location, structure, substrate specificity and physiological roles, PLA_2_s can be classified into secreted PLA_2_s (sPLA_2_s), cytosolic PLA_2_s (cPLA_2_s), calcium-independent PLA_2_s (iPLA_2_s), and lysosomal-associated PLA_2_s (lPLA_2_s). These PLA_2_ isoforms are similar in that they can all cleave cellular membrane-associated phospholipids, releasing free lysophospholipids and fatty acids such as arachidonic acid, which subsequently serve as precursors for a wide range of bioactive mediators responsible for physiological functions and pathological changes. Respiratory infections, especially those caused by bacteria and viruses, represent a substantial threat to the health of the population worldwide and cause billions of disease cases and millions of deaths annually. Respiratory infections provoke airway inflammation, characterized by increased vascular permeability and the influx of immune cells, resulting in tissue damage, impaired gas exchange, acute respiratory distress syndrome (ARDS) and even death. During infections and inflammatory milieu, airway-expressed PLA_2_ can further increase and exhibit protection by restricting pathogens and inflammation or, in contrast, exacerbate the pathogenesis. In this manuscript, we will provide an overview of the current knowledge on the biological functions of PLA_2_ isoforms, especially concerning membrane-associated isoforms in respiratory infections, and offer insight into the spatial and temporal regulation of immune responses mediated by PLA_2_ and the subsequent modulation of host–pathogen interactions and the balance between protective effects and pathological outcomes.

## 1. Introduction

In addition to cytokines and chemokines [1], which have been intensively studied, lipid signaling, especially when mediated by polyunsaturated fatty acids (PUFAs) or short-chain fatty acids (SCFAs), has emerged as a critical regulator of early inflammation [2,3]. Lipid mediators influence immune cell recruitment, epithelial barrier integrity, and antimicrobial effector functions, often acting within minutes of infection and thus being critical for early immune response. Central to the generation of these lipid signals is the phospholipase A_2_ (PLA_2_) superfamily, the members of which catalyze the hydrolysis of membrane phospholipids to release free fatty acids like arachidonic acid (AA), oleic acid (OA) [4] and lysophospholipids (LPLs). These fatty acids and their metabolites contribute to the regulation of crucial cellular processes such as cell proliferation, migration, survival, and differentiation [5]. Through the generation of these lipid mediators, the PLA_2_ superfamily plays a vital role in cellular homeostasis, immune signaling, and host defense, while dysregulation of its enzymes is linked to inflammation, cardiovascular diseases, cancer, and neurodegeneration [6,7,8].

Respiratory infections are the leading cause of global morbidity and mortality, driven by pathogens that exploit the lungs’ unique structural and immunological environment [9,10]. Effective host defense in the respiratory tract depends not only on pathogen recognition but also on the timing, magnitude, and localization of early immune responses, which can determine whether inflammation promotes microbial control or progresses toward tissue damage and chronic disease [11,12]. The airway epithelium constitutes one of the largest mucosal surfaces in the body, in which immune components need to deal with substantial amounts of exogenous microbes and restrict the invasion of diverse pathogens. Despite its enrichment in airway epithelial cells, as well as alveolar and interstitial macrophages, the immunomodulatory role of PLA_2_ in lung immunity has long been underappreciated [13]. In support of its critical role, PLA_2_ dysregulation was found to cause excessive inflammation in bacterial and viral pneumonia, while appropriately tuned activity of PLA_2_ may support protective immunity [14]. This balance is particularly relevant in chronic respiratory infections such as tuberculosis (TB), wherein early immune events shape long-term disease trajectories such as fibrosis, persistent lung inflammation and reduced lung capacity [15,16]. These data highlight the continuous impact of PLA_2_ signaling initiated during the acute infection.

Despite increasing acknowledgement of PLA_2_ enzymes as regulators of lipid mediator production during pulmonary inflammation, the exact role of PLA_2_s in lung immunity remains elusive, partly due to isoforms with distinct cellular localizations and functions, particularly in human lung tissue [17,18]. Despite recent efforts to identify specific roles for PLA_2_ isoforms in lung pathology, including in epithelial injury, fibrosis, and immune responses during infection [19,20,21], how PLA_2_ activity is regulated across distinct lung compartments such as the airway epithelium, alveolar space, and infection-associated structures, including granulomas, has not been systematically characterized [22,23]. In addition, infection-induced altered lipid metabolism, including lipid droplet accumulation and surfactant remodeling, is also associated with PLA_2_-dependent lipid signaling [24,25], which adds complexity to the task of clarifying the contribution of PLA_2_ to lung immunity. Notably, much of the current understanding of PLA_2_ function in the lungs is derived from isolated experimental systems, whereas direct comparisons across infection models remain limited. As a result, it is still unclear whether observed effects reflect universal biological mechanisms or model-specific responses, underscoring the need for more integrative and context-aware analyses.

Therefore, we believe that a review of the current progress in PLA_2_ investigation fits the current demands and will benefit ongoing and future studies. In this context, we will focus on the role of PLA_2_ isoforms in respiratory infections, highlighting that their spatial and temporal regulation influences early lung immunity, host–pathogen interactions, and the balance between protection and pathology.

## 2. Overview of PLA_2_ Superfamily

### 2.1. General Function and Classification of PLA_2_ Enzymes

**Figure 1:**
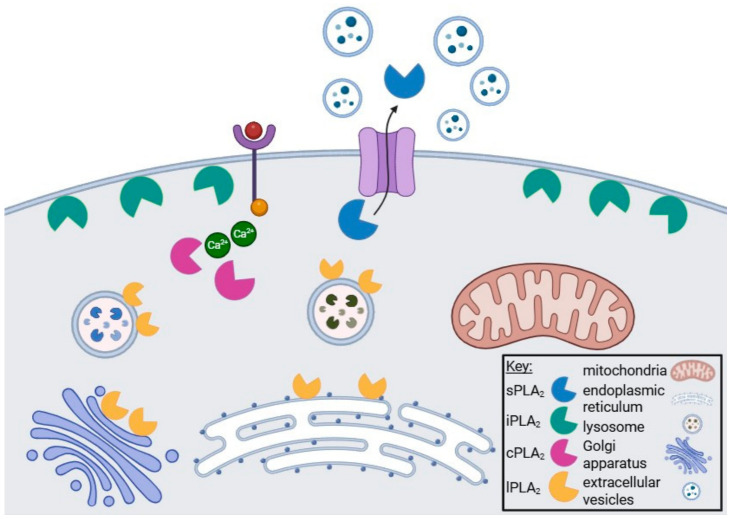
Locations of PLA_2_ isoforms. Created in BioRender. Walter, K. (2026) https://BioRender.com/hdgfb4x (accessed on 22 April 2026).

The PLA_2_ superfamily constitutes a diverse range of lipid-hydrolyzing enzymes that catalyze the cleavage of the sn-2 ester bond of membrane phospholipids, resulting in the release of free fatty acids such as AA and LPL. Despite these shared catalytic activities, individual PLA_2_ family members differ in their subcellular localization, structure, and calcium dependence (Figure 1), which leads to distinct biological functions. Based on their characteristics, mammalian PLA_2_s are broadly classified into secreted PLA_2_ (sPLA_2_), cytosolic PLA2 (cPLA_2_), calcium-independent PLA_2_ (iPLA_2_), and lysosomal-associated PLA_2_ (lPLA_2_), with the latter three isoforms being located mainly in cytosol [26,27]. While this classification provides a useful biochemical framework, functional distinctions between these isoforms in immune settings are poorly defined, particularly in the milieu of lung biology.

**Figure 2:**
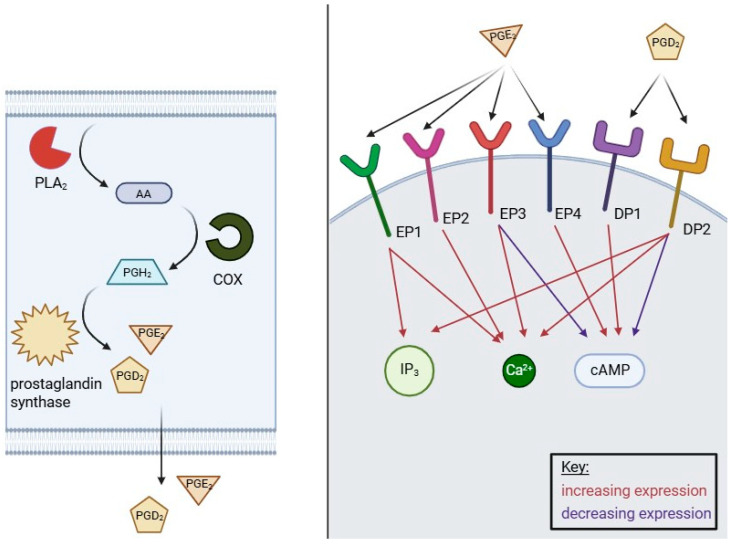
Differentiated PLA_2_ signaling. Created in BioRender. Walter, K. (2026) https://BioRender.com/hdgfb4x (accessed on 22 April 2026).

Lipid products generated by PLA_2_ signaling serve as precursors for a wide range of bioactive mediators through cyclooxygenase-, lipoxygenase-, and cytochrome P450-mediated pathways, including eicosanoids, platelet-activating factors, and LPL-derived signaling molecules, thereby positioning PLA_2_ at the intersection of membrane biology, inflammation, and cell signaling [17,24,28]. These subclasses are not redundant; rather, they act at different cellular sites and contexts to coordinate membrane remodeling, inflammatory signaling, and host defense [18,29,30]. In addition to the complexity of the specific and overlapping functions mediated by distinct lipid mediators produced by PLA_2_ signaling, some of them may exhibit a wide spectrum of effects by binding specific receptors. For example, prostaglandin D2 (PGD_2_) shows anti-inflammatory function by binding PTGDR (also known as DP1) expressed by myeloid cells, but can promote pro-inflammatory response mediated by type 2 T cells through the CRTH2 receptor (also known as DP2) [31]. Similar issues have also been identified in PGE_2_ signaling, which can be differentiated via at least four different receptors (Figure 2).

### 2.2. Lipid Substrate Specificity and Triggering of PLA_2_ Activity

PLA_2_ isoforms exhibit distinct substrate preferences and regulatory constraints during hydrolyzing of glycerophospholipids embedded within biological membranes. For example, cPLA2α (group IVA), displays a strong preference for phospholipids containing AA at the sn-2 position, making it a key upstream regulator of eicosanoid biosynthesis during inflammatory responses [27,32]. In contrast, iPLA_2_ isoforms are less selective with respect to fatty acid composition and are considered to function primarily in membrane phospholipid remodeling and homeostasis under less-stressed conditions [33,34]. In addition, the activity of PLA_2_ isoforms, even sPLA_2_, is tightly anchored to the biological membrane. Rather than acting on soluble substrates, these enzymes interact dynamically with lipid bilayers, sensing changes in membrane curvature, composition, and integrity, features that are frequently altered during pathogen entry, phagocytosis, or intracellular replication [6]. During infections, viruses actively interact with host cell membranes to obtain their entry by membrane fusion (in enveloped viruses), by transient local disruption of membrane integrity (in most non-enveloped viruses), or by cell lysis to facilitate their replication [35,36,37]. On the other hand, many bacterial pathogens invade target cells via interaction with the host cell membrane via the zipper or trigger mechanisms, leading to the activation of a wide range of membrane-associated pathways like vesicle trafficking, which may help them escape from phagocytosis [38,39]. These alterations in membrane dynamics during infection trigger the activity of PLA_2_s, which subsequently provide regulatory signals as feedback.

### 2.3. Secreted vs. Membrane-Associated PLA_2_

PLA_2_ secretion is primarily driven by activation of the innate immune system, inflammatory disorders with increased intracellular calcium levels, and membrane interactions [40,41,42]. sPLA_2_s are typically released into extracellular spaces or luminal compartments, where they can hydrolyze microbial membranes or modify extracellular lipid mediators, resulting in bacterial killing. The antimicrobial properties of sPLA_2_a in host defense, especially at mucosa surfaces, have been well established [28]. Work from Murakami and colleagues has further shown that distinct sPLA_2_ isoforms act in a context-dependent manner and contribute to immune regulation in tissue-specific settings [18,27]. For example, sPLA_2_-IIA has been associated with antibacterial defense and the amplification of inflammatory responses through hydrolysis of bacterial and host membrane phospholipids, whereas sPLA_2_-V and sPLA_2_-X more prominently regulate phospholipid remodeling, macrophage activation, and eicosanoid generation in airway and allergic inflammation models. These studies further showed that individual sPLA_2_ isoforms display distinct substrate preferences and cellular localization patterns, allowing them to differentially influence leukocyte recruitment, epithelial signaling, and tissue-specific immune responses rather than functioning as uniform amplifiers of inflammation. These findings highlight the need for and feasibility of developing context-specific intervention strategies. Nevertheless, the origin and potential isoforms of sPLA_2_s, especially their communication with membrane-associated PLA_2_s, deserve further investigation.

Unlike sPLA_2_s, membrane-associated PLA_2_s, especially cPLA_2_ and iPLA_2_, are uniquely positioned and act at intracellular membranes and organelles, which allow them to directly couple pathogen sensing with lipid mediator production and downstream responses, including the modulation of intracellular signaling and membrane dynamics that influences phagosome maturation, antigen processing, and immune cell activation. These features make them particularly relevant in chronic or intracellular infections, where host defense depends on coordinated immune regulation rather than direct microbial killing. Similarly to cPLA_2_, lPLA_2_ was first identified in lysosomes and primarily functions at intracellular membranes. Despite their original locations, these isoforms can be activated by cellular stimuli and be translocated to specific membrane compartments, such as the plasma membrane, nuclear envelope, endosomes, mitochondria, and lysosomes. Their intracellular localization allows them to coordinate lipid signaling with downstream pathways including cytokine production, inflammasome activation, autophagy, and cell death [26,43]. In addition, many pathogens, especially intracellular bacteria and viruses, exploit host membranes during entry, replication, and egress. Perturbations in membrane structure and lipid composition serve as danger signals that can be sensed by membrane-associated PLA_2_s, leading to localized lipid mediator production and activation of innate immune pathways [44] (https://www.balsinde.org/publists/matlab2.pdf accessed on 30 April 2026). Due to dynamic changes and communication concerning the membrane structure, as well as the translocalization of PLA_2_, advanced tracking techniques are warranted to clarify the unique role of those isoforms.

## 3. Membrane-Associated PLA_2_ Isoforms

### 3.1. Cytosolic PLA_2_α

cPLA_2_s usually exhibit calcium-dependent activity, while cPLA_2_α represents the most extensively characterized member of the PLA_2_ superfamily and plays a central role in inflammation-driven lipid signaling. Upon elevations in intracellular calcium and phosphorylation by Mitogen-activated protein kinases (MAPKs), cPLA_2_α can be translocated from cytosol to perinuclear membranes, the plasma membrane, and endosomal compartments [27]. This spatial redistribution enables localized AA release and subsequent eicosanoid production, resulting in a rapid integration of receptor-mediated signals in immune and epithelial cells [18,45,46]. In these cells, cPLA_2_α activation is closely linked to receptor-mediated signaling pathways, including Toll-like receptors (TLSs) and cytokine receptor activation, positioning this enzyme as a key amplifier of inflammatory responses [32].

### 3.2. Calcium-Independent PLA_2_β and PLA_2_γ (Group VIA)

iPLA_2_β and iPLA_2_γ represent major isoforms of iPLA_2_ and are ubiquitously expressed and operate independently of intracellular calcium fluctuations. These enzymes are constitutively active and have been found to regulate basal phospholipid turnover, membrane repair, and organelle integrity [33]. iPLA_2_β is usually localized at the cytosol and endoplasmic reticulum where it is implicated to regulate immune cell survival, cytokine production, and signal amplification downstream of pattern recognition receptors (PRRs), while iPLA_2_γ is enriched in mitochondria and peroxisomes where it likely has a specialized role in organelle-specific lipid metabolism, influencing organelle integrity and redox balance [34]. While iPLA_2_ activity has been linked to cell survival, mitochondrial function, and modulation of inflammatory signaling in immune cells, its role in host–pathogen interactions remains less well defined compared to cPLA_2_α [47,48].

### 3.3. Lysosomal PLA_2_ (PLA_2_G15/lPLA_2_)

Lysosomal PLA_2_, encoded by the *pla2g15* gene, is localized predominantly at acidic compartments such as lysosomes and late endosomes. Unlike other PLA_2_ isoforms, PLA_2_G15 exhibits optimal activity at low pH and contributes to phospholipid degradation within lysosomal membranes [49]. Emerging evidence suggests that this enzyme plays a role in phospholipid degradation during membrane turnover and lipid antigen processing and presentation in macrophages and dendritic cells, linking lysosomal lipid metabolism with adaptive immune activation [50,51]. Given that many intracellular pathogens reside within or are manipulated in endo-lysosomal compartments, PLA_2_G15 serves as a potential but underexplored mediator at the membrane–pathogen interface [28].

In summary, the functional diversity of membrane-associated PLA_2_s is largely dictated by their precise subcellular localization and regulatory mechanisms. Based on the defined membrane regions of PLA_2_-mediated activities, lipid mediators are generated locally and can rapidly influence nearby signaling events. This feature allows PLA_2_ isoforms to integrate environmental cues, such as pathogen-derived signals or inflammatory stimuli, with tightly regulated lipid mediator production at specific intracellular sites of immune and epithelial cells. As for translational development, differential targeting of the plasma membrane or endosomal, mitochondrial, or lysosomal compartments enables compartment-specific lipid signaling responses [26,27].

## 4. PLA_2_ in Respiratory Infections

### 4.1. PLA_2_ in Lung Homeostasis

Membrane-associated PLA_2_s, particularly cPLA_2_α and selected sPLA_2_ isoforms, occupy an important position at the interface of membrane biology and innate immune regulation in the lungs. In airway epithelial cells and alveolar macrophages, engagement of PRRs during infections activates MAPK pathways and induces intracellular calcium flux. These signals promote phosphorylation-dependent translocation of cPLA_2_α to perinuclear membranes, where it hydrolyzes membrane phospholipids and releases AA from the sn-2 position [52,53]. This enzymatic step represents a major regulatory checkpoint in lipid mediator generation. The release of AA leads to the production of prostaglandins (PGs) and leukotrienes (LTs), shaping the local cytokine environment and modulating leukocyte responses, suggesting that membrane-associated PLA_2_ enzymes function as modulators of immune tone rather than simple amplifiers of inflammation.

Under steady-state lung conditions, low levels of PLA_2_ activity help maintain immune balance in an accessible environment that is constantly exposed to inhaled particles and exogenous antigens. Basal expression of prostaglandin E2 (PGE_2_), the downstream product of cytosolic PLA_2_ signaling, helps maintain alveolar macrophages in a restrained activation state. This lipid-mediated signaling elevates intracellular cyclic adenosine monophosphate (cAMP) and limits excessive nuclear factor-kappa B (NF-kB)-driven transcription, thereby preventing inappropriate cytokine release while preserving the capacity to respond to danger signals [54,55]. Recent lung-focused immunology studies further emphasized that metabolic programming and lipid regulation are central determinants of macrophage identity, epithelial–immune cross-talk, and tissue repair dynamics in the steady-state lung environment [56,57,58,59]. In particular, the metabolic state of alveolar macrophages, which is shaped by lipid-rich surfactant components and local nutrient availability within the alveolar niche, influences their differentiation, activation thresholds, and capacity to maintain immune tolerance [56,59]. Meanwhile, release of polyunsaturated fatty acids provides substrates for the generation of specialized pro-resolving mediators such as lipoxins and resolvins. These mediators enhance efferocytosis, support epithelial repair, and contribute to the restoration of barrier integrity after minor tissue stress [60]. Through these interconnected pathways, membrane-associated PLA_2_ activity helps maintain a balance between immune vigilance and tissue protection that is central to pulmonary homeostasis.

Beyond mediator biosynthesis, PLA_2_ also drives membrane remodeling and influences the structural and metabolic landscape of innate immune cells. Changes in phospholipid composition can alter lipid raft organization and thereby affect clustering of TLRs and the intensity of their downstream signaling [53]. In alveolar macrophages, shifts in membrane lipids intersect with mitochondrial function and reactive oxygen species (ROS) generation, processes that dominate inflammasome priming and cytokine maturation. iPLA_2_ isoforms also participate in phospholipid turnover within endo-lysosomal compartments, thereby indirectly affecting antigen handling and basal cytokine thresholds [48]. In addition, lipid-dependent signaling pathways support bidirectional communication between airway epithelial cells and resident immune populations. Airway epithelial cells regulate macrophage behavior through the release of cytokines, alarmins, and lipid mediators, while macrophages in turn influence epithelial barrier integrity and coordinate repair processes following injury [57,58]. Metabolic-lipid signaling networks therefore play an important role in maintaining pulmonary immune homeostasis while preserving the ability of the lungs to mount rapid and appropriately regulated responses to inhaled pathogens and environmental stimuli [57,59].

Although membrane-bound PLA_2_ isoforms are increasingly recognized as regulators of lung homeostasis, their precise roles remain difficult to reconcile across studies [6,53,61]. Several reports suggest that baseline PLA_2_ contributes to immune tolerance in the steady-state lung by sustaining low-level production of immunoregulatory lipid mediators. In murine models of allergic airway inflammation and lung injury, cPLA_2_-dependent generation of PGE_2_ has been shown to restrain excessive macrophage and dendritic cell activation through cyclic AMP-dependent signaling pathways, thereby limiting NF-κB-driven inflammatory responses. In parallel, PLA_2_-mediated release of polyunsaturated fatty acids provides substrates for the generation of specialized pro-resolving mediators such as lipoxins and resolvins, which promote efferocytosis, suppress neutrophil accumulation, and support epithelial repair during the resolution phase of lung inflammation [60,62]. However, the extent to which this activity actively enforces tolerance versus simply reflects ongoing membrane turnover remains unclear, as direct in vivo evidence is limited. Addressing this distinction is critical, as it remains unclear whether PLA_2_ activity represents a conserved regulatory mechanism or a highly dynamic response shaped by inflammatory context. In addition, while lipid-dependent metabolic programming has been implicated in shaping alveolar macrophage identity and epithelial–immune cross-talk, much of this evidence derives from steady-state or in vitro systems, with limited validation in infection-relevant contexts [54,57,63]. This raises an important question as to whether the same PLA_2_-driven pathways that support homeostasis are preserved, amplified, or fundamentally reprogrammed during early infection, particularly given the dynamic changes in cellular composition and inflammatory signaling in the infected lung [63,64].

### 4.2. PLA_2_ in Host Defense and Inflammation

Through the generation of lipid mediators, PLA_2_ plays a critical role in infection-induced immune activation and inflammatory resolution [17,18]. During respiratory infections (Table 1), PLA_2_-mediated signaling becomes more dynamically engaged and contributes to the early inflammatory milieu that shapes host responses to invading pathogens. Both secreted and membrane-associated PLA_2_ isoforms participate in this process, with membrane-associated ones playing a critical role in coordinating localized cellular responses within infected lung tissues [17].

#### 4.2.1. Context-Dependent Roles of cPLA_2_α

Activation of cPLA_2_α has been reported across multiple models of respiratory viral infections, particularly in those caused by influenza and respiratory syncytial virus. In these settings, cPLA_2_α-mediated AA release drives downstream eicosanoid production, resulting in promoting inflammatory signaling, recruiting innate immune cells, and modulating epithelial and dendritic cell function during early infection. Despite their potential protective effects, excessive activation of these pathways can also amplify lung inflammation and contribute to tissue damage during severe infection [72]. Evidence suggests that cPLA_2_α may also influence inflammasome activation through its regulation of lipid mediator production and membrane phospholipid composition. In addition, cPLA_2_α-driven changes in membrane lipid composition may influence the assembly of inflammasome complexes, but direct mechanistic evidence in pulmonary infection models remains limited. Consistent with that, AA-derived eicosanoids have been implicated in modulating NLRP3 inflammasome signaling, although the reported effects are context-dependent and vary across experimental systems [18,53].

In bacterial infections such as TB, cPLA_2_α activity is similarly linked to macrophage activation and inflammatory signaling pathways that influence granuloma formation and disease progression. However, the impact of cPLA_2_α can vary across stages of infection, the composition of immune cells within the lesion, and the overall inflammatory environment. During early infection, cPLA_2_α-mediated AA release can support macrophage activation and promote the production of lipid mediators that contribute to antimicrobial responses and immune cell recruitment [17,22]. As infection progresses and granulomas develop, changes in cellular composition, including the accumulation of macrophages, neutrophils, and lymphocytes, can alter the balance of lipid mediators produced within the tissue [18,22]. In more advanced or highly inflammatory lesions, sustained cPLA_2_α activation may instead amplify eicosanoid-driven inflammation, which can cause tissue damage, necrosis, and altered granuloma stability [17,73]. These stage-dependent effects highlight how PLA_2_-mediated lipid signaling can exert both protective and pathological roles during infections, setting the timeframe for appropriate intervenes.

#### 4.2.2. iPLA_2_ Modulates Membrane Stability and Metabolism

iPLA_2_ isoforms play a central role in phospholipid remodeling, which is critical for maintaining membrane integrity during infection-induced stress. This function is closely linked to mitochondrial homeostasis and cellular metabolism, as the disruption of membrane lipid composition can impair oxidative phosphorylation and promote metabolic dysfunction [18,48]. In macrophages, such metabolic alterations may influence the balance between pro-inflammatory and reparative phenotypes, although direct evidence linking iPLA_2_ activity to infection-induced metabolic reprogramming in the lung remains limited. The iPLA_2_ isoforms iPLA_2_β and iPLA_2_γ both contribute to respiratory host defense primarily through roles in membrane maintenance and cellular stress adaptation. During infection-induced stress, these enzymes support mitochondrial and plasma membrane integrity, thereby sustaining immune signaling and cell viability [48]. Upon exposure to viral or bacterial pathogens, infection often induces oxidative stress, mitochondrial dysfunction, and membrane damage in immune and epithelial cells. Under these conditions, iPLA_2_-dependent phospholipid turnover helps preserve mitochondrial function and cellular metabolism, which are necessary for maintaining antimicrobial responses and cytokine signaling [18,74]. Disruption of iPLA_2_ activity has been associated with altered susceptibility to respiratory pathogens, likely reflecting impaired immune cell survival and stress adaptation rather than direct modulation of inflammatory mediator production [24,48].

#### 4.2.3. lPLA_2_ Contributes to Intracellular Pathogen Manipulation

lPLA_2_ was found to contribute to the host defense, particularly in respiratory infections caused by intracellular pathogens, through its role in lysosomal phospholipid turnover and phagolysosome maturation. lPLA_2_ dominates in the lysosomal phospholipid degradation process, which is essential for phagolysosome maturation and efficient processing of intracellular pathogens [68,69]. During *Mycobacterium tuberculosis* (Mtb) and *Legionella pneumophila* infections, efficient phagosome maturation and lysosomal function are critical for restricting intracellular pathogen survival within macrophages. Mtb actively interferes with phagolysosome fusion and prevents acidification of infected compartments, thereby avoiding lysosomal degradation and enabling long-term persistence within host macrophages. Similarly, *L. pneumophila* remodels host vesicular trafficking pathways to generate replication-permissive vacuoles that evade lysosomal maturation. Although direct studies on lPLA_2_ deficiency in pulmonary infection models remain limited, impaired lysosomal phospholipid turnover would be expected to compromise membrane remodeling, phagolysosome stability, and antigen processing, potentially reducing intracellular pathogen clearance and adaptive immune activation [22,75]. By regulating phospholipid degradation within lysosomes, lPLA_2_ also modulates the membrane remodeling and antigen processing pathways that support the presentation of microbial antigens to T cells [24,51,69].

### 4.3. PLA_2_ and Bacterial Respiratory Infections

#### 4.3.1. Acute Bacterial Lung Injury and Pneumonia

Bacterial pneumonia remains a major cause of global morbidity and mortality, with pathogens such as *Streptococcus pneumoniae* and *Pseudomonas aeruginosa* frequently implicated. Effective host defense requires rapid coordination of innate immune mechanisms, including phagocyte activation, cytokine release, and neutrophil recruitment to the infected lung.

PLA_2_ signaling is activated early in response to bacterial components such as lipoteichoic acid and lipopolysaccharide (LPS), leading to the generation of lipid mediators that promote neutrophil migration and inflammatory amplification [17,76]. sPLA_2_ isoforms, particularly groups IIA and V, are induced in lung epithelial cells and alveolar macrophages during pneumonia and contribute to surfactant hydrolysis, bacterial membrane disruption, and propagation of inflammatory signals [77]. Human sPLA_2_-IIA functions as a bactericidal enzyme against group B streptococcus (GBS), making it crucial for host protection against systemic infection and lung challenge by GBS [78]. Despite its pathogen-eliminating capability, excessive sPLA_2_ activity may degrade pulmonary surfactants and contribute to alveolar collapse, edema, and impaired gas exchange. In support of that, experimental evidence demonstrates that PLA_2_ inhibitors significantly lower the severity of cellular damage in LPS-induced lung injury in a mouse model [21].

Membrane-associated PLA_2_, especially cPLA_2_α, function upstream of eicosanoid biosynthesis in macrophages and neutrophils. Activation of cPLA_2_α downstream of TLR signaling promotes AA release and subsequent leukotriene and prostaglandin synthesis, which coordinate early chemotaxis and effector functions [6,30,79]. Experimental models demonstrate that genetic deletion or pharmacologic inhibition of cPLA_2_α attenuates neutrophil recruitment and inflammatory mediator production, highlighting its central role in bacterial lung inflammation [52,80]. An in vivo study revealed *P. aeruginosa*-induced mortality is increased by cPLA_2_α activation, which causes excessive IL-6 production through 15-lipoxygenase (15-LOX), cyclooxygenase-2 (COX-2), and MAPK–extracellular-signal-regulated kinase (ERK)/p38) pathways [81]. However, in a study of *C. Albicans*-induced lung infection, cPLA_2_α aids in innate immune defense mechanisms to control the infection and dampen inflammation. cPLA_2_α^−/−^ mice showed excessive inflammation with increased recruitment of neutrophils and the production of pro-inflammatory cytokines, and a 10-fold increase in IL-6 levels compared to cPLA_2_α^+/+^ mice. Therefore, the role of cPLA_2_α in pulmonary infection is pathogen-dependent, acting to either enhance or mitigate infection-driven injury [82]. iPLA_2_ also contributes to antibacterial responses by regulating membrane remodeling and phagosome function. These processes are particularly relevant during infection with intracellular or phagosome-associated bacterial pathogens such as *Mycobacterium tuberculosis* and *Legionella pneumophila*, wherein macrophage membrane dynamics and phagosome maturation are critical determinants of pathogen control [75]. Although its roles remain less well characterized than those of cPLA_2_, emerging evidence suggests that iPLA_2_ activity can influence macrophage metabolic adaptation and inflammatory output during bacterial challenge, thereby shaping the host response to infection [18,21]. Lastly, lPLA2 has been implicated in lysosomal phospholipid turnover and phagolysosome membrane remodeling, processes that may influence intracellular bacterial processing, antigen presentation, and lysosome-dependent microbial killing. These functions are likely to be particularly relevant for intracellular pathogens, which evade phagolysosome maturation to survive within macrophages [24,68].

Collectively, these findings indicate that PLA_2_ pathways are integral to early innate immune orchestration during acute bacterial respiratory infections. However, because lipid mediators can also drive tissue injury, the magnitude and timing of PLA_2_ activation are critical determinants of whether inflammation promotes pathogen clearance or pathological lung damage.

#### 4.3.2. Tuberculosis

Tuberculosis (TB), caused by Mtb, is characterized by chronic granulomatous inflammation and an elaborate balance between host defense and immunopathology [83,84]. Unlike acute pneumonia, TB involves sustained immune activation, metabolic reprogramming, and localized tissue remodeling over prolonged periods. Host lipid metabolism is a prominent feature of TB pathology, with lipid droplet accumulation in infected macrophages contributing to the formation of foamy macrophages within granulomas [22]. These lipid stores provide substrates for both bacterial metabolism and host-derived lipid mediators that influence immune outcomes. The PLA_2_, cPLA_2_α and sPLA_2_ isoforms, in particular, are well positioned to regulate this environment by controlling AA availability and LPL generation within infected lungs [23,85]. sPLA_2_ acts as a critical mediator of Mtb-induced inflammation in microglial cells, which subsequently signals through the classical protein kinase C (PKC) family or the Ras/Raf-1/MEK/ERK pathway the stimulation of ROS generation and cytokine release [86]. The in vitro role of cPLA_2_ in Mtb infection is contradictory. Although cPLA_2_-IVA expression is induced by Mtb in mouse bone marrow-derived macrophages (BMDMs), either pharmacological inhibition of PLA_2_ or infection in cPLA_2_-IVA-deficient BMDMs does not alter intracellular bacterial survival in comparison to wild-type cells. [87]. Conversely, other research indicates that TNF-mediated activation of cPLA_2_ drives the apoptosis of Mtb-infected human macrophages, serving as a protective host defense mechanism [88]. These findings highlight potential differences in host-specific antimycobacterial mechanisms with distinct bacteriostatic and bactericidal activities, in which human macrophage-expressed PLA_2_s may act redundantly with cPLA_2_-IVA, thereby masking its function. Based on its unique production by macrophages, lPLA_2_ exhibits a protective role in host immunity to TB. lPLA_2_ absence impairs alveolar macrophage functions in vivo leading to defective T-cell priming and deficient CD4+ and CD8+ T-cell recruitment to infected lungs, along with lower Th1-type cytokine levels, which establishes the beneficial role of lPLA_2_ in triggering adaptive T-cell immunity to Mtb [14].

In addition, animal studies implicate PLA_2_-dependent leukotriene pathways in modulating neutrophil recruitment and inflammatory balance during TB, with altered lipid mediator signaling influencing both bacterial control and immunopathology [89]. Human studies similarly associate elevated PLA_2_ activity and eicosanoid signatures with disease severity, suggesting that dysregulated lipid signaling may favor pathological inflammation rather than effective containment [90,91]. PLA_2_-driven lipid mediator networks also intersect with cytokine pathways central to TB-induced immunity, including IL-1 and TNF. These interactions form feedback loops that can either support granuloma integrity or exacerbate tissue damage depending on the inflammatory context [85]. Taken together, these studies demonstrate the long-term impact of PLA_2_ signaling in the disease progression of TB. With the identification of post-acute sequelae caused by diverse respiratory pathogens, it is intriguing to determine the contribution of PLA_2_ signaling to those conventional “acute infections” and their aftermath in an extended timeline.

### 4.4. PLA_2_ and Viral Respiratory Infections

Respiratory viral infections like Influenza, Severe acute respiratory syndrome coronavirus (SARS-CoV) and SARS-CoV-2 inequitably cause higher morbidity and mortality in elderly people compared to younger populations. Diverse age-dependent defects have been identified in the airway, including oxidative stress and inflammaging (chronic low-grade inflammation) [92,93,94,95,96], and result in increased severity upon infection as well as less effective development of immune memory after vaccination or natural infection [97,98]. Upon aging-related stress, the expression level of PLA_2_s, of group IID (PLA_2_G2D) in particular, is increased in lung epithelial cells and myeloid cells, especially dendritic cells [31,99]. The elevation of lung PLA_2_ subsequently increases the production of lipid mediators like PGD_2_, PGE_2_, PGF_2_α, and thromboxane, which exhibit both pro- and anti-inflammatory properties by amplifying cytokine production and vascular leak, or directly regulating host immune response induced by infections [31], contributing to acute respiratory distress syndrome (ARDS)-like pathology [100].

#### 4.4.1. Influenza and Other Orthomyxoviruses

Influenza A virus (IAV) infection elicits rapid innate immune responses in the lung, with early cytokine and chemokine production strongly influencing disease severity. PLA_2_ signaling-generated lipid mediators, including PGs and LTs, contribute to both protective antiviral immunity and inflammatory pathology during infection [100].

cPLA_2_α activation in influenza-infected epithelial cells and macrophages drives eicosanoid production that affects neutrophil recruitment and vascular permeability [17,101]. In mouse models, inhibition of cPLA_2_α reduces lung inflammation and improves survival, although excessive suppression can impair viral clearance, underscoring its context-dependent role [100]. Similarly, Zhao et al. reported an age-dependent increased expression of PGD_2_, a downstream product of PLA_2_G2D (either membrane-bound or secreted), in mice which was directly proportional to the decrease in respiratory DC (rDC) migration upon Influenza infection. PLA_2_G2D-PGD_2_/DP1 signaling deficiency (by establishing *Pla2g2d^−/−^* and *ptgdr^−/−^* mice) or blockade (by using specific antagonists) enhances respiratory DC migration from infected lungs to draining lymph nodes (DLNs), subsequently enhancing virus-specific T-cell responses and improving mice survival [102]. On the other hand, 15-Deoxy-prostaglandin J2 (15d-PGJ2), a metabolite of PGD_2_, was reported to significantly decrease severe influenza morbidity and mortality via activation of the peroxisome proliferator-activated receptor-gamma (PPARγ) pathway, which reduces the exaggerated lung pro-inflammatory response [103]. Recent studies also implicate sPLA_2_ isoforms in shaping adaptive immune responses during influenza infection. For example, *Pla2g2e^−/−^* mice with a lack of secreted PLA_2_G2E had significantly lower survival rates and higher viral loads in their lungs compared to wild-type mice. Although the authors claimed that PLA_2_G2E selectively modulates adaptive T-cell immunity without affecting innate or humoral defense [104], the mechanisms underlying T-cell modulation remain elusive.

#### 4.4.2. CoV Infection and Severe Viral Pneumonias

Severe human pathogenic CoV infections, including SARS-CoV, SARS-CoV-2 and Middle East respiratory syndrome (MERS)-CoV, are characterized by exaggerated inflammatory responses characterized by elevated cytokines and lipid mediators. Increased levels of PGs, LTs, and related metabolites have been detected in the lung tissue and circulation of patients with severe COVID-19 [65]. Although CoVs do not encode PLA_2_ enzymes, CoV infections can perturb host membrane systems and activate innate immune pathways that engage PLA_2_ signaling.

cPLA_2_α activity is crucial in replication organelle (RO) formation during human CoV (HCoV)-299E and MERS-CoV replication in vitro. Pharmacological inhibition of cPLA_2_α using a specific small-molecule inhibitor, pyrrolidine-2 (Py-2) drastically reduces the formation of double-membrane vesicles (DMVs) and DMV-associated viral replication/transcription complexes, which confirms its role in CoV RNA synthesis [105]. An elevated level of sPLA_2s_ is also correlated with COVID-19 severity and acute multisystem inflammatory syndrome (MIS-C) in children, suggesting their role in inflammasome activation and disease pathogenesis [106]. Marked elevations in six circulating isoforms of sPLA_2_ (sPLA_2_-IIA, sPLA_2_-V, sPLA_2_-X, sPLA_2_-IB, sPLA_2_-IIC, and sPLA_2_-XVI) was found in deceased COVID-19 patients [107,108]. In support of that, Snider et al. found that the levels of circulating sPLA_2_-IIA closely mirrored multiple indicators of disease severity, including hyperglycemia, kidney dysfunction, hypoxia, anemia, and multiple organ dysfunction [108].

Same as its role in influenza virus infection, the PLA_2_G2D signaling pathway and its lipid mediators PGD_2_ and PGE_2_ possess significant roles in COVID-19 infections. Ghimire et al. reported that COVID-19 disease enhancement in IL-13-enhanced mice is mediated by the PLA_2_G2D-eicosanoid signaling pathway [109]. Increased expression of PLA_2_G2D is reported to be directly proportional to disease severity in mice [102]. SARS-CoV, SARS-CoV-2 and MERS-CoV infection leads to an increase in PGD_2_ levels in both mice and humans [110,111]. The elevated levels of PGD_2_ inhibits inflammasome activation by inhibiting IL-1β signaling through its DP1 receptor [110]. SARS-CoV and MERS-CoV infection initiates binding of PGD_2_ on the DP1 receptor which results in diminished antiviral responses by hindering rDC migration to the lymph nodes via inhibition of CCR7 upregulation. This decrease affects Langerhans and regulatory DCs, which in turn diminishes T-cell responses. Defects in rDC migration occur progressively as mice age and the degree of impairment may vary according to pathogens [31,112,113]. However, the anti-inflammatory profile of PLA_2_G2D-PGD_2_/DP1 signaling has a bright side. Although PLA_2_G2D deficiency protects hosts from lethal infections, convalescent mice show impaired production of virus-specific antibodies. Mechanically enhanced T-cell responses (mainly type 1 and 17 T cells) identified in *Pla2g2d^−/−^* mice are generated at the cost of decreased follicular helper T (Tfh) cells, which are critical for the induction of B-cell memory and antibody production. Blocking IL-1β signaling produced by rDC in *Pla2g2d^−/−^* mice reverses the impaired Tfh and antibody-producing B cells [31,110]. These data serve as critical justification for further investigating the long-term impact mediated by PLA_2_ signaling and carefully addressing the efficacy and potential side effects of PLA_2_-targeting therapies.

**Figure 3:**
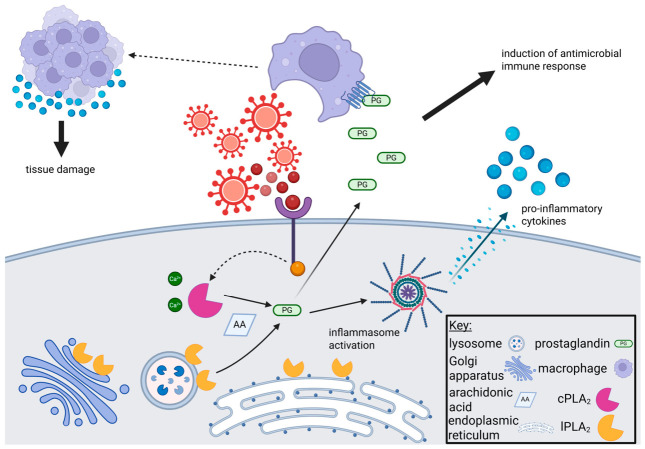
Dual facets of PLA_2_-mediated effects during infections. Created in BioRender. Walter, K. (2026) https://BioRender.com/hdgfb4x (accessed on 22 April 2026).

The role of PGE_2_ was also intensively investigated in COVID-19. Researchers found that SARS-CoV-2 infection induces PGE_2_ generation and secretion in infected lung epithelial cells via TNF-α expression, which increases COX-2 expression and decreases PGE_2_ degrading enzyme hydroxy prostaglandin dehydrogenase (HPGD) expression [114]. In addition, IgG specific for the CoV spike protein also contributes to the production of PGE_2_ in non-polarized (M0) and in M1 and M2-type polarized human macrophages in the presence of a D-dimer [115]. Subsequently, PGE_2_ promotes inflammatory response in macrophages in the lungs and associated lymph nodes, contributing towards disease severity. This cascade then impairs both the innate and adaptive immune response by decreasing the release of cytokines and impairing the proliferation, activation and survival of T cells and B-cell responses [114]. Thus far, it has been shown that PGE_2_ has immunosuppressive capabilities which can enhance disease severity [116,117]. Elevated PGE_2_ could induce hyperinflammation and immune responses during COVID-19 infection [114,118]. The diverse effects of PGE_2_ are due to its heterogeneity in its coupling to its receptors EP1, EP2, EP3, and EP4 and receptors downstream to intracellular signaling cascades. EP receptors mediate production of cAMP or the cell entrance of Ca^2+^ causes pain, inflammation, mitogenesis, and cell injury [119]. Persistent high levels of PGE_2_ with imbalanced EP3 receptor activity also plays a pivotal role in the development of long COVID [120]. Emerging clinical and experimental data suggest similar mechanisms operate in COVID-19, with PLA_2_ activity correlating with disease severity and adverse outcomes [108]. These findings point to shared lipid signaling pathways across viral pneumonias, with membrane-associated PLA_2_s acting as upstream regulators of inflammatory lipid mediator production (Figure 3).

#### 4.4.3. Other Respiratory Virus Infections

Respiratory syncytial virus (RSV) is a common respiratory virus that infects the nose, throat, respiratory tract, and lungs. Compared to healthy adults, young kids and especially infants are susceptible to severe bronchiolitis induced by RSV infection and can develop persistent asthma [121]. As mentioned above, PGD_2_ also signals through DP2 and enhances Th2-mediated immune responses, such as asthma [122]. Consistent with that, an elevated levels of PGD_2_ induced by IFN-λ were identified in RSV-infected young infants and may contribute to the development of asthma. In support of this, the severe RSV bronchiolitis in an RSV-infected neonate mouse model can be blocked by a DP2 inhibitor, resulting in decreased viral load, immunopathology, and morbidity. Overall, enhancement of PGD_2_ levels would be beneficial for respiratory viral infections and antivirals targeting PGD_2_ signaling pathway may be useful for the treatment of respiratory infections.

## 5. PLA_2_-Targeting Therapy in Treating Respiratory Disease

The growing recognition of PLA_2_ signaling as a regulator of lipid-mediated immune responses has greatly enhanced interest in corresponding targeting therapies, although the isoform-specific and context-dependent functions of this signaling remain to be clarified before their clinical application. Recent studies highlight cPLA_2_α as a central node in inflammatory lipid signaling, with newer work emphasizing its role in infection-associated inflammation and immune dysregulation rather than simply eicosanoid production [123]. While pharmacological inhibition of PLA_2_-associated pathways remains feasible, clinical benefit has been inconsistent, underscoring the complexity of targeting upstream lipid metabolism [124].

The targeting of downstream pathways, including cyclooxygenase and lipoxygenase, continues to be a widely used method in airway disease, but these approaches lack isoform specificity and fail to address upstream lipid remodeling [125,126]. Emerging lipidomic studies in respiratory infections, particularly COVID-19, further demonstrate extensive remodeling of phospholipid and eicosanoid pathways that correlate with immune dysregulation and disease severity [100,127].

Together, these findings suggest that PLA_2_ signaling functions as a dynamic regulator of host responses rather than conserved and static targets. Effective therapeutic strategies will therefore require isoform-selective approaches with precise temporal control, as early inhibition may impair host defense, whereas later modulation may help limit tissue damage. Here we include a list of PLA_2_-targeting treatments in use or in clinical/pre-clinical trials (Table 2).

## 6. Questions to Be Answered

Despite the promising potential of PLA_2_-targeting therapy in treating respiratory infectious diseases, some basic questions remain to be clarified before clinical application.

In recent years, diverse agonists and antagonists targeting PLA_2_-mediated signaling have been developed. However, successful clinical trials are still absent, which may be caused by multiple factors, including species differences, choice of administration protocols, and most importantly, our limited knowledge about these signaling pathways.

As described above, PLA_2_-mediated signaling may exhibit both pro-inflammatory and pro-resolving effects under different contexts, including different phases of infections. The underlying mechanisms contributing to this functional switch need to be further investigated in appropriate models.

In addition to their functions, the positions of PLA_2_s at the interface of membrane dynamics, lipid mediator production, and innate immune signaling make them critical determinants of disease trajectory. Clarifying the mechanisms regulating the distribution of PLA_2_s between different positions may provide a novel strategy to modify PLA_2_-mediated signaling.

The structure and function overlaps of PLA_2_-AA metabolic products make it challenging to identify the specificity of individual signaling. A deeper understanding of isoform-specific functions may open new avenues for host-directed therapies in respiratory infections.

Finally, although aging- and stress-related increases in PLA_2_-mediated signaling have been widely identified in animal models and human patients, the driving forces of these increases, especially the metabolism of the microenvironment and the dynamics of microbiota, remain to be illustrated in future studies.

## Figures and Tables

**Table 1:** PLA2 Signaling Responses to Respiratory Infections.

PLA_2_ Isoform	Cellular Source	Key Mechanism	Infection Context/Pathogens	Protective Role	Pathological Role	Evidence Type	Key References
cPLA_2_α	Airway epithelial cells, alveolar macrophages	Arachidonic acid (AA) release and downstream eicosanoid production	Influenza virus, RSV, SARS-CoV-2, *Mycobacterium tuberculosis*	Promotes antiviral responses, regulates leukocyte recruitment	Excess eicosanoid production drives inflammation and tissue damage	In vitro, in vivo, clinical	[18,53,65]
iPLA_2_β/γ	Macrophages, epithelial cells	Phospholipid remodeling, mitochondrial integrity, metabolic adaptation	*M. tuberculosis*, *Streptococcus pneumoniae*, *Influenza virus*	Maintains membrane homeostasis and immune cell viability	Dysregulation impairs immune function and stress responses	In vitro, limited in vivo	[48,66,67]
LPLA_2_ (PLA2G15)	Lysosomes of macrophages	Lysosomal phospholipid degradation, phagolysosomal maturation, antigen processing	*M. tuberculosis*, *Legionella pneumophila*	Supports intracellular pathogen clearance and antigen presentation	Role in pathology remains poorly defined	Mechanistic, limited infection models	[68,69]
sPLA_2_ (selected isoforms)	Secreted from epithelial cells and immune cells	Extracellular phospholipid hydrolysis, modulation of lipid signaling	Viral and bacterial pneumonia	Modulates immune tone and host defense responses	Can amplify inflammation depending on context and isoform	In vitro, in vivo	[18,64,70,71]

**Table 2:** PLA_2_ inhibitors.

PLA2 Inhibitor	Target PLA2	Indication/Disease	Clinical Trial Phase/Status	Major Outcome	Reference
Varespladib (LY315920)	sPLA2	COVID-19, sepsis, acute inflammatory disorders	Phase II for COVID-19	Reduced sPLA2 activity and inflammatory mediators; COVID-19 trial terminated early due to slow enrollment;	ClinicalTrials.gov, NCT04969991
Varespladib methyl (LY333013)	sPLA2	Acute coronary syndrome and systemic inflammation	Phase III	Lowered circulating sPLA2 levels but failed to improve clinical cardiovascular outcomes	[128]
Darapladib	L-PLA2	Atherosclerosis, coronary artery disease	Phase III	Inhibited Lp-PLA2 activity but did not significantly reduce major cardiovascular events	[129]
Rilapladib	L-PLA2	Alzheimer’s disease	Phase II	Inhibited Lp-PLA2 activity with possible potential to slow down the progression of Alzheimer’s disease	[130]
AK106-001616	cPLA2	Anti-Inflammatory/analgesic drug, rheumatoid arthritis	Phase II	Reduced inflammatory lipid mediators (prostaglandins and leukotrienes) with better GI profile compared to naproxen	[131]
Giripladib (PLA-695)	cPLA2	Osteoarthritis	Phase II	Terminated due to GI issues and a lack of significant superiority over existing treatments	ClinicalTrials.gov, NCT00396955
ZPL-5212372	cPLA2	Topical application for atopic dermatitis, previously tested with inhaled route for asthma	Phase I/II	Drug found to be safe and well tolerated	ClinicalTrials.gov, NCT02795832
LY3127760	EP4 receptor	Inflammatory disease	Phase I	Inhibited PGE2 signaling and demonstrated anti-inflammatory pharmacodynamic activity	ClinicalTrials.gov, NCT01968070
Vipoglanstat	mPGES-1 inhibitor	Systemic sclerosis-related Raynaud’s phenomenon	Phase II	Reduced PGE2 levels but was ineffective in systemic sclerosis	[132]
LY3023703	mPGES-1 inhibitor	Inflammatory disorders	Phase I	Inhibited PGE2 synthesis by >90%	[133]

## Data Availability

No new data were created or analyzed in this study. Data sharing is not applicable to this article.
